# Fractured Lateral Humeral Condyle Accompanied by Acute Radial Nerve Palsy in a Child: First Reported Case in the United Arab Emirates

**DOI:** 10.7759/cureus.37772

**Published:** 2023-04-18

**Authors:** Baher Mohamed Samy, Ahmed Hafez Mousa, Mohamed Mostafa Ali Mohamed, Bilal El Yawafi

**Affiliations:** 1 Department of Orthopedics and Trauma Surgery, Dubai Health Authority, Rashid Hospital, Dubai, ARE; 2 College of Medicine and Surgery, Batterjee Medical College, Jeddah, SAU

**Keywords:** orif, trauma and orthopedic surgery, radial nerve injury, fractured lateral humeral condyle, pediatric orthopedic surgery

## Abstract

Despite being very common, lateral condyle fractures in children are rarely associated with acute nerve injuries. We present the case of a 10-year-old, left-handed male child who presented with a left lateral humeral condyle fracture associated with radial nerve injury. The patient was managed by open reduction and internal fixation plus radial nerve exploration, which was found entrapped in the fracture site. The patient achieved full recovery after 16 weeks. We report this case to present the approach and the operative findings and to emphasize the importance of a preoperative clinical examination in addition to preoperative planning to achieve a favorable outcome.

## Introduction

Lateral humeral condyle fractures are relatively common in children, but the associated radial nerve palsy is a rare complication [1]. The radial nerve runs in close proximity to the lateral humeral condyle, making it susceptible to injury [2,3]. In children, lateral humeral condyle fractures are one of the most common types of elbow injuries, accounting for up to 19% of all pediatric elbow fractures [4,5]. Here, we report a rare occurrence of acute radial nerve paralysis after a fractured lateral humeral condyle in a child.

## Case presentation

**Figure 1:**
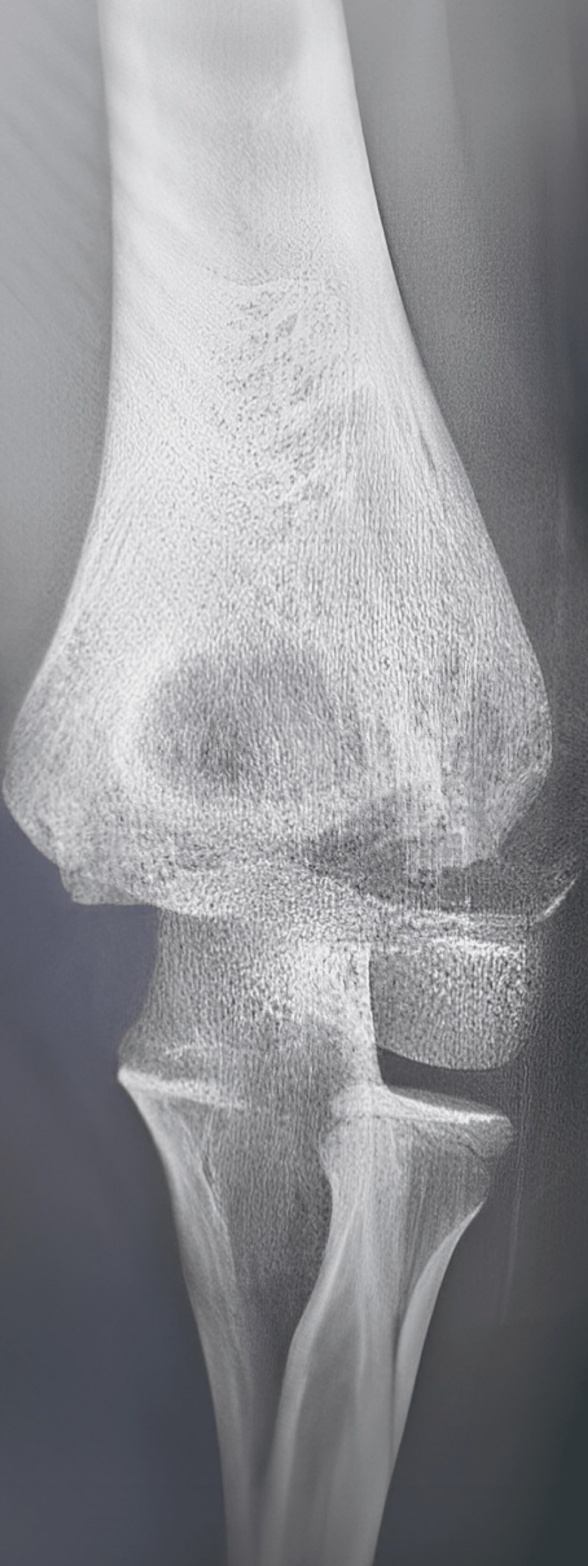
An X-ray was taken of the affected area, which showed a left lateral humeral condyle fracture with displacement

We present the case of a 10-year-old, left-handed male child who presented to our emergency department with left elbow pain, swelling, and inability to move it. He gave a history of falling down on an outstretched hand while playing football. Further detailed examination showed that he was unable to actively extend the wrist, fingers, and thumb. Sensory examination showed diminished sensation over the radial nerve territory. The motor and sensory functions of the ulnar and median nerves were normal. A detailed routine neurological examination was performed. There was a complete loss of active left wrist extension, an active extension of the fingers at the level of the metacarpophalangeal joint, as well as a loss of active extension of the thumb. Sensory examination using light touch and pinprick showed sensory loss affecting the lateral 3½ digits and the associated area on the dorsum of the hand. He had palpable distal pulses. X-rays were requested and showed a Milch TYPE II fracture of the lateral humeral condyle (Figure 1).

A diagnosis of fractured left lateral humeral condyle associated with acute radial nerve palsy was established.

Operative intervention was decided by open reduction and internal fixation plus radial nerve exploration. Pre-anaesthesia clearance was obtained, and consent was obtained from the parents after clearly explaining the nature of the injury, the planned surgical intervention, and the possible operative findings, including possible radial nerve laceration and the need for repair. 

Surgery was done under general anesthesia and a tourniquet. The anterolateral approach to the elbow was chosen over the formal lateral approach usually used in lateral condyle fractures because we assumed it will offer better radial nerve exploration and visualization.

**Figure 2:**
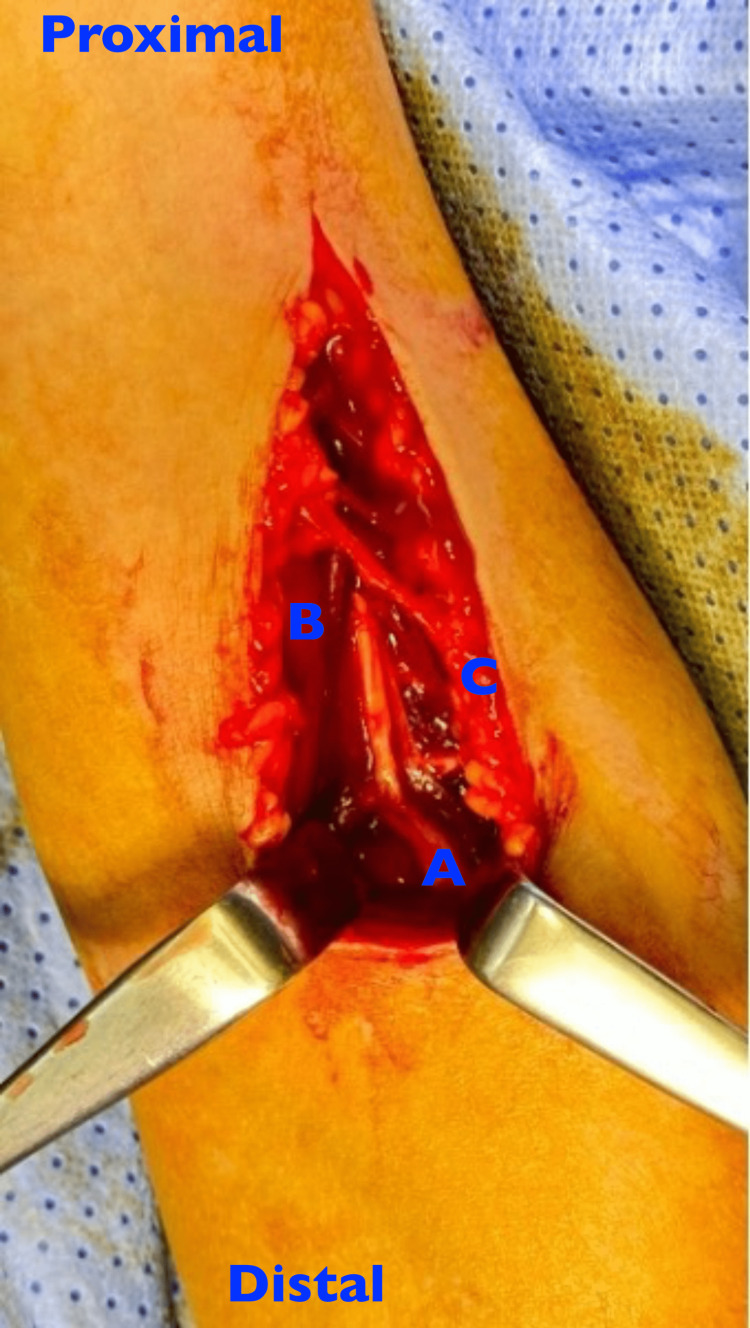
At the level of the fracture the radial nerve was found entrapped between the fracture ends (A) Radial nerve entrapped at the fracture site, (B) Brachialis muscle, (C) Brachioradialis muscle

After skin and subcutaneous dissection. The interval between the brachialis and brachioradialis muscles was developed well above the fracture, and the radial nerve was identified and carefully followed distally. At the level of the fracture, the radial nerve was found entrapped between the fracture ends (Figure 2).

**Figure 3:**
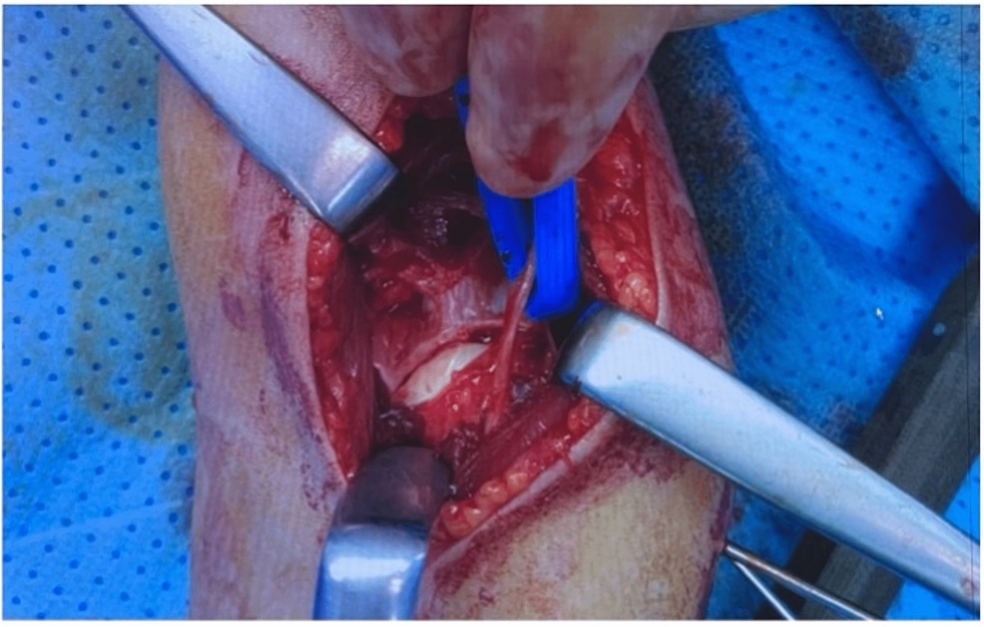
The nerve was meticulously released to avoid further injury and was found contused but continuous and without lacerations

The nerve was meticulously released to avoid further injury and was found contused but continuous and without lacerations (Figure 3).

**Figure 4:**
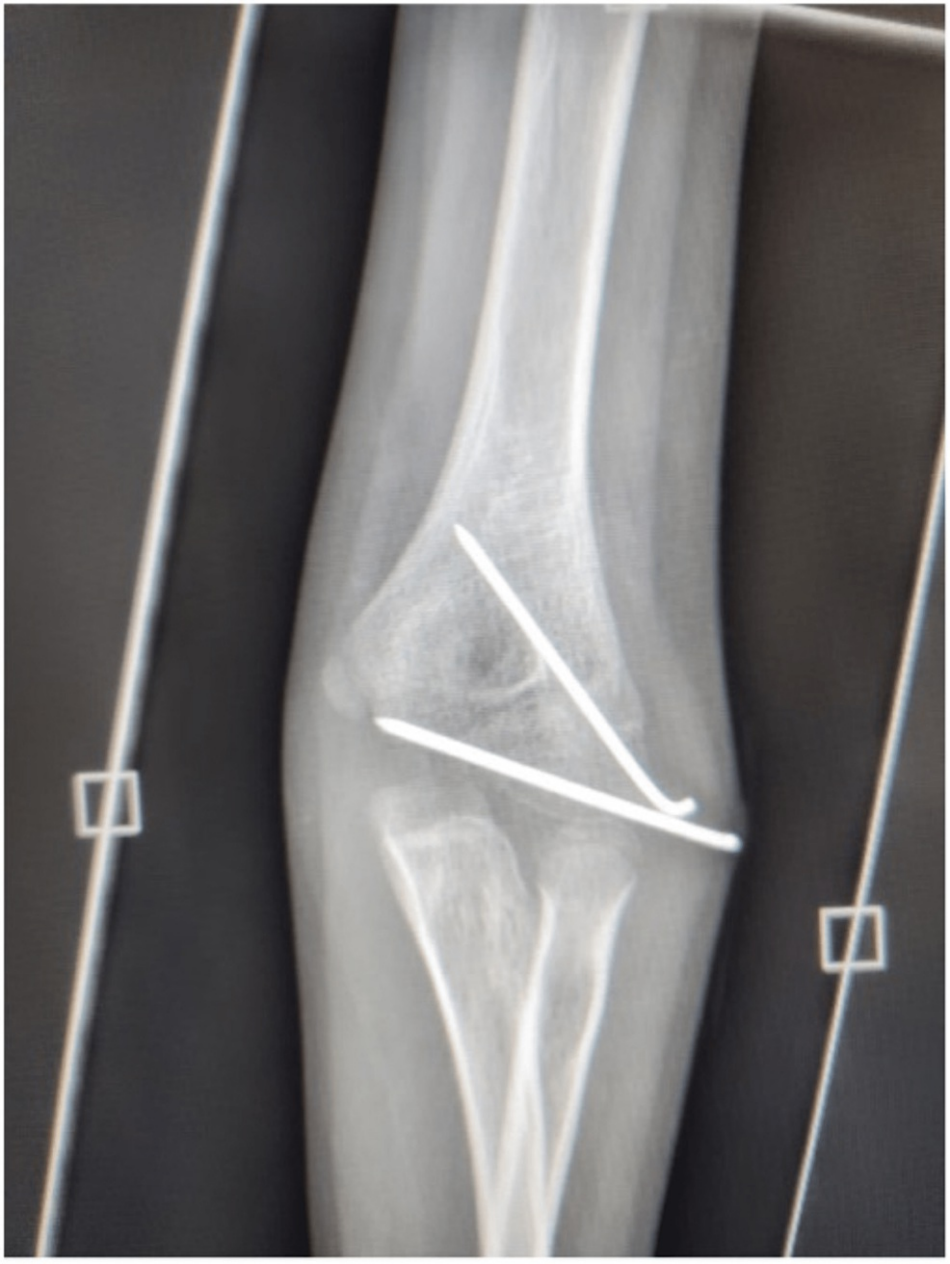
During the six-week follow-up appointment, X-rays were taken, which showed that the lateral humeral condyle fracture had achieved solid union

Further attention was paid to the fracture, which was reduced anatomically after clearing the hematoma and was fixed by two 2 mm K-wires; one K-wire was used to fix the articular surface and the other was used to fix the lateral condyle to the metaphysics. The K-wires' position was confirmed under fluoroscopy. The tourniquet was released, and hemostasis was achieved. The K-wires were bent, cut, and kept under the skin. The wound was closed in layers with the skin closed with absorbable sutures. Dressing and an above-elbow slab with a cockup were applied. Postoperatively, the patient was examined again and showed no recovery of the radial nerve. He was discharged with instructions and given a follow-up appointment. At the six-week follow-up, X-rays showed a solid union of the fracture (Figure 4).

**Figure 5:**
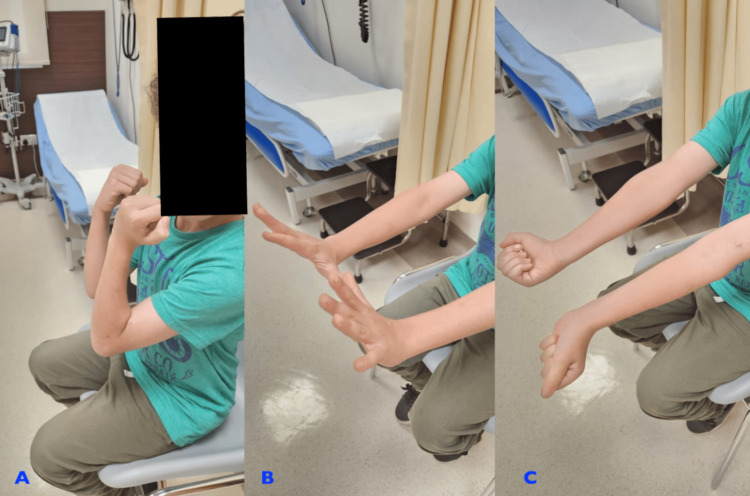
After 16 weeks of postoperative recovery, the patient's range of motion in the elbow was fully restored and the radial nerve has fully recovered (A, B, C)

**Figure 6:**
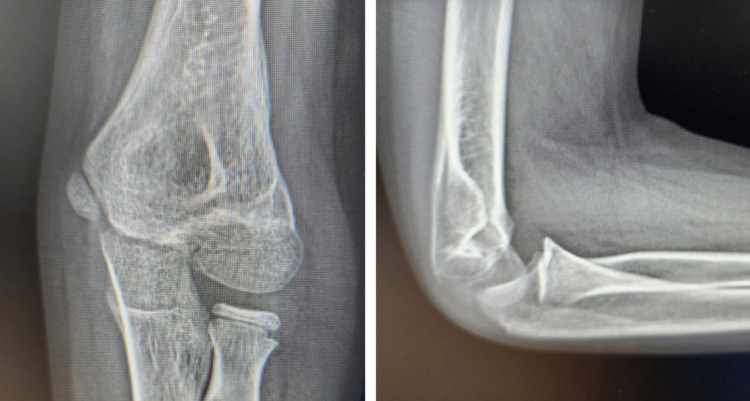
At 16 weeks postoperatively, the fracture had fully healed and the K-wires were removed

The slab was removed, and the patient was allowed to start active elbow range of motion exercises. Neurological examination showed improvement in the numbness and partial recovery of the wrist extension; however, the fingers and thumb drops persisted. K-wires were removed at eight weeks postoperatively under local anesthetic. At 12 weeks, the patient had full recovery of the wrist and finger extension and only the thumb drop persisted. At 16 weeks, the patient had complete recovery of the thumb extension and retained a good elbow range of motion of 0-150 degrees (Figures 5-6).

## Discussion

Radial nerve paralysis associated with a lateral humeral condyle fracture in children is a rare but potentially serious injury [6]. A displaced middle and distal humeral fracture often causes radial nerve palsy [7,8]; however, radial nerve palsy accompanied by a lateral humeral condyle fracture is rare in both children and adults [8-10]. Proper management starts with a prompt diagnosis. It is assumed that the neurologic assessment is not adequate when children are injured. Mayne reported that only 12 of 137 children with supra-condylar fractures had a complete preoperative neurologic or vascular assessment documented [11]. A preoperative full neurological assessment and documentation are of paramount importance to guide the management plan and choice of surgical approach and to avoid the legal issues of a claimed iatrogenic injury during fixation. In similar cases, radial nerve function returned with appropriate treatment of exploration, the release of the radial nerve when required, and internal fixation of the fracture [11]. The “push-off” and “pull-off” theories have been proposed as possible mechanisms for lateral humeral condyle fractures. We believe that the fracture was caused by a push-off force. Push-off means a valgus and axial force on the lateral condyle by the impacted radial head during falling on an outstretched hand. According to the pull-off theory, if the injury had been caused by avulsion force, the lateral condyle fragment should have moved laterally, and it would have been difficult to cause direct injury to the radial nerve. On the other hand, the push-off theory proposes that valgus and axial compression both combined with an element of hyperextension would force the lateral condyle toward a posterolateral direction while causing stretching of the radial nerve, consequently resulting in a fracture and subsequent radial nerve palsy [11]. Although the push-off mechanism would force the lateral condyle in a posterolateral direction away from the nerve, we believe that in this case, the radial nerve was stretched over the proximal fragment of the fracture and got caught under the sharp edge of the humerus metaphyses and eventually got entrapped. Table 1 summarizes previous studies reporting on radial paralysis with left lateral epicondyle fractures. We assume that the anterolateral approach is appropriate in such cases as it allows direct visualization and careful release of the radial nerve without causing further injuries.

**Table 1:** Summary of previous studies reporting on radial paralysis with lateral humeral condyle fractures

Reference	Study Type	Country	Age (years)	Gender	Number of cases	Treatment	Recovery Time (months)
Nemoto et al. [8]	Case report	Japan	6	Female	1	Open reduction and internal fixation	6
Muthulingam et al. [10]	Case report	India	13	Male	1	Open reduction and internal fixation	3

## Conclusions

In conclusion, acute radial nerve palsy accompanying a fractured lateral humeral condyle is a very rare condition in children. We present this case to highlight the importance of a detailed neurological clinical examination in every case to avoid missing rare injuries. We also present the rare operative findings and prognosis of this injury.
